# Projected long-term impacts of US funding cuts on TB and HIV in South Africa and the TB Programme response

**DOI:** 10.5588/ijtldopen.26.0165

**Published:** 2026-10-12

**Authors:** M. Kubjane, L. Jamieson, L.F. Johnson, J. Boffa, P. Subrayen, F. Abdullah, N. Ndjeka, L. Lebina, P. Naidoo, E. Mohr-Holland, G. Meyer-Rath

**Affiliations:** 1 Health Economics and Epidemiology Research Office (HE^2^RO), University of the Witwatersrand, Johannesburg, South Africa;; 2 South African TB Think Tank, Johannesburg, South Africa;; 3 Centre for Integrated Data and Epidemiological Research (CIDER), University of Cape Town, South Africa;; 4 The Aurum Institute, Johannesburg, South Africa;; 5 Office of AIDS and TB Research, South African Medical Research Council, Cape Town, South Africa;; 6 Departments of Public Health Medicine & Infectious Diseases, University of Pretoria, Pretoria, South Africa;; 7 TB Cluster, South African National Department of Health, Pretoria, South Africa;; 8 Africa Health Research Institute, Durban, South Africa;; 9 Community Services and Health, City of Cape Town, Cape Town, South Africa;; 10 Boston University School of Public Health, Boston, MA, USA.

**Keywords:** HIV-associated tuberculosis, antiretroviral therapy, public health, global health funding

Dear Editor,

South Africa has made great strides in reducing TB, with TB incidence and mortality dropping by 30.4% and 47.7%, respectively, between 2009 and 2019, primarily driven by expanded testing and antiretroviral therapy (ART) scale-up.^1^ Despite these gains, the country carries one of the highest burdens of TB and TB-HIV co-infection worldwide. In 2024, we estimated approximately 320,000 new TB episodes and 62,000 deaths, half occurring among people living with HIV (PLHIV).^2^ In early 2025, the momentum of these gains was threatened by the US government’s withdrawal of funding. In 2024/2025, South Africa’s National TB Programme (NTP) was largely domestically funded (67%), with external funding from the Global Fund (19%), and the USAID and PEPFAR (14%), while PEPFAR funded 14% of the HIV programme.^3,4^

Following our report on the immediate impacts of US funding cuts on TB services and research in South Africa,^5^ the NTP tasked us with estimating the potential long-term consequences for TB incidence and mortality under scenarios in which these funding reductions are unmitigated. Additionally, we provide an update on the NTP’s response to the funding cuts. This report aims to inform urgent policy and financing decisions by quantifying the potential long-term consequences of service disruptions and highlighting the scale of the remaining recovery efforts required to prevent the reversal of hard-won gains in TB prevention and care. We used the Thembisa TB/HIV mathematical model, which combines TB and HIV epidemic dynamics and interventions (prevention, testing, and treatment).^1,2,6^ We modelled intervention reduction scenarios informed by NTP stakeholder consultations,^7^ providing plausible intervention reduction ranges as minimum and maximum scenarios (Table), which were triangulated with expenditure data.^4^ Scenarios assumed varying reductions in TB preventive therapy, testing, and treatment linkage. HIV service scenarios varied ART coverage only, given its strong effect in reducing progression to active TB disease and TB-related deaths among PLHIV.^8,9^ We estimated additional TB cases and deaths under four scenarios without additional resources to close the US funding gap: minimum–maximum reductions without recovery (2025–2035) and minimum–maximum reductions with gradual recovery to baseline levels starting in 2029, corresponding to the end of the current US administration’s term.

**Table.** Stakeholder-informed assumptions regarding reductions in TB and HIV programme intervention coverages.

Baseline	Minimum	Maximum
TB programme interventions		
TB preventive therapy for people living with HIV	20%	25%
TB testing and screening (outreach, finding vulnerable clients, collecting sputum samples)	20%	25%
Linkage to TB treatment	15%	25%
HIV programme interventions		
Antiretroviral treatment coverage	14%	28%

ART coverage reductions of 14%–28% alone increased new HIV infections (2025–2035) by 235,000 (16%) with recovery, and up to 1,000,000 (67%) without recovery (Figure panel A). The resulting higher HIV incidence and lower ART coverage drove a 12%–41% rise in HIV-associated TB episodes and increase in 21%–72% deaths among PLHIV. We projected additional TB cases ranging from 223,000 (7%) in the ‘minimal impact with recovery’ scenario to 731,000 (24%) in the ‘maximal impact with no recovery’ scenario (Figure panel B). The mortality impact would be even more severe: relative to the baseline estimate of 481,000 TB deaths between 2025 and 2035, additional deaths could range from 67,000 (14%) in the ‘minimal impact with recovery’ scenario to 225,000 (47%) in the ‘maximal impact without recovery’ scenario (Figure panel B). Recovery scenarios would require an additional 8–12 years to return TB incidence and mortality to baseline trajectories, highlighting the long-lasting consequences of even temporary service disruptions.

**Figure.**
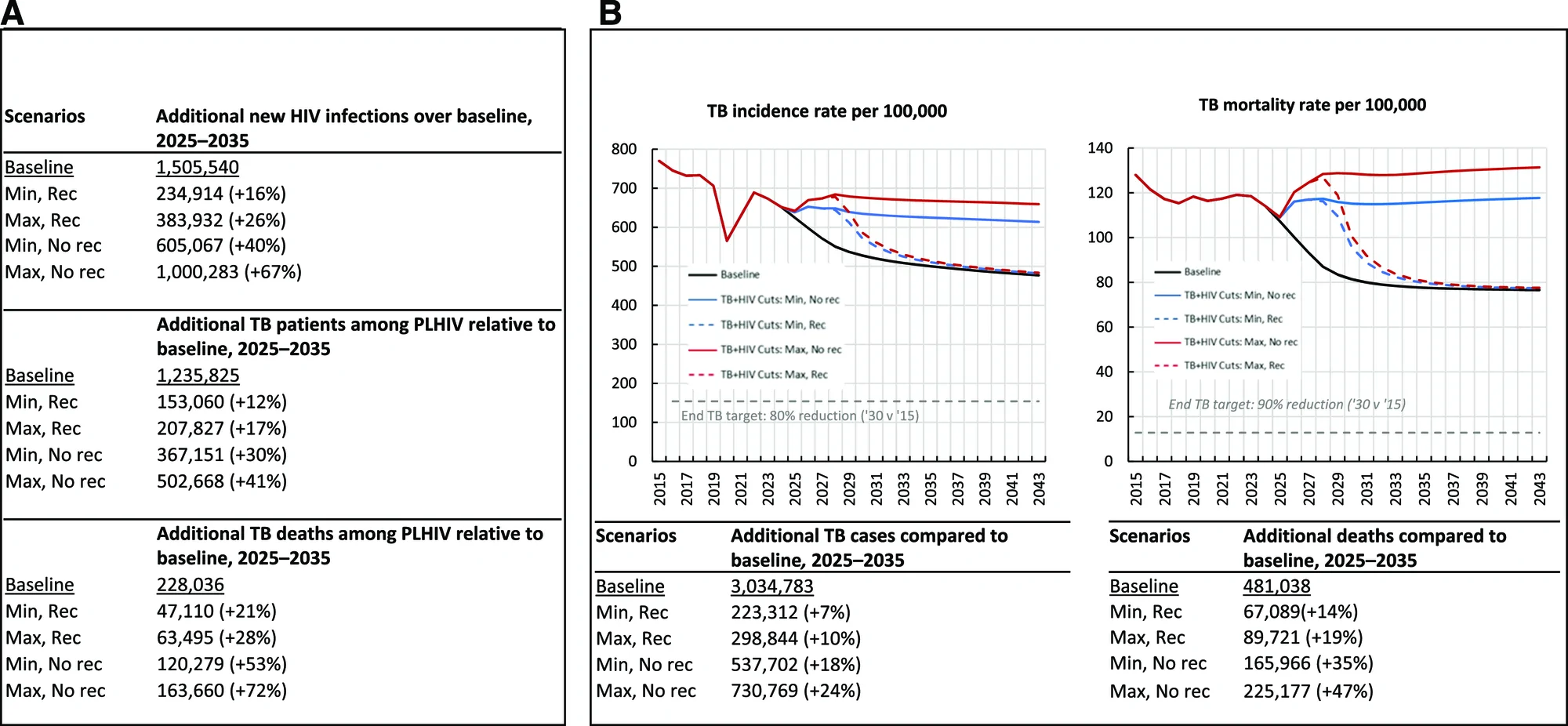
Projected increases in HIV incidence, HIV-associated TB, and overall TB incidence and mortality, 2025–2035. **A:** Impact on new HIV infections and HIV-associated TB incidence and mortality; **B:** Combined impact of TB and HIV programme disruptions on population-level incidence and mortality. PLHIV = people living with HIV; Max = maximum; Min = minimum; Rec = recovery.

The South African NTP has responded to mitigate the impact of these funding cessations through annual TB Recovery Plans that prioritised TB diagnosis, linkage, and retention in care.^4,10^ One of the key priorities of the 2025/26 TB Recovery Plan, linked to the End TB Campaign, was to test 5 million people for TB. During the 2025 calendar year, approximately 3.56 million tests were conducted.^4,10^ Although testing reached only 71% of the target, the campaign likely offset the projected 20%–25% (600,000–800,000) reduction in testing capacity under the modelled funding disruption scenarios. Furthermore, in 2025, the South African government announced an allocation of an additional US$ 41.7 million in emergency funds to mitigate the impact of the withdrawal of PEPFAR funding.^11,12^ Of this amount, US$ 32 million has been allocated to provinces through the District Health Program grant for comprehensive HIV services, and US$ 7.1 million to the South African Medical Research Council for HIV research. However, the specific allocation to TB programmes is unclear.

Building on our initial report,^5^ we modelled the long-term impacts of unmitigated funding disruptions, projecting catastrophic consequences on TB incidence and mortality. In the worst-case scenario, without service recovery, there could be an additional 731,000 TB cases and 225,000 deaths, erasing more than a decade of progress. These disruptions have interrupted the momentum of the NTP’s efforts to scale up preventive treatment, community-based tracing, and targeted TB testing – still mid-scale-up, and most relying on US-funded partners.^4,13^ Even with complete service restoration in 2029, it could take 8–12 years to return to baseline trends. Highlighting that delayed responses to these funding disruptions could have long-lasting consequences, amplifying TB illness and lives lost. Additionally, disruptions to treatment linkage would likely worsen treatment uptake and outcomes, increasing the likelihood of drug resistance.^13^ Projections include a 12%–41% increase in additional cases and 21%–72% in TB deaths among PLHIV, highlighting the integrated nature of the TB and HIV epidemics in this high-burden setting. It also demonstrates how disruptions to HIV services have profound consequences for TB. ART scale-up remains one of the most effective interventions for preventing TB in South Africa,^14^ and any reversal of ART coverage will unsurprisingly drive increases in HIV-associated TB and the overall burden of disease.

The South African government’s allocation of US$ 41.7 million^11,12^ to mitigate the funding gaps represents an important step towards domestic resource mobilisation. However, transparency on funds ring-fenced for TB is required to strengthen accountability. Additionally, the funding crisis presents an opportunity to explore alternative financing mechanisms, such as social impact bonds.^15^ The End TB Campaign^4,10^ demonstrates what coordinated action and political will can achieve. Sustaining these efforts requires efficient and strategic implementation – including innovations such as near-point-of-care tests (e.g., PlusLife MiniDock MTB) to reduce diagnostic costs; and, in the longer term, effective TB vaccines. Their impact will depend on coordinated planning, financing, and integration into adequately functioning TB and HIV programmes.

Given the NTP’s additional funding and increased testing, our projections of the impact of US funding cuts are pessimistic. Nonetheless, these results are important in illustrating the potential impact without mitigation efforts. Additionally, the setbacks highlighted here have implications beyond South Africa’s national targets, threatening regional and global End TB Strategy milestones, and also highlight fragility of NTPs reliant on external donors. Sustained political commitment, transparent resource allocation, and protection of core TB and HIV services remain essential to prevent reversals in progress toward TB elimination.
